# Blended Care Interventions to Promote Physical Activity: A Systematic Review of Randomized Controlled Trials

**DOI:** 10.1186/s40798-022-00489-w

**Published:** 2022-07-30

**Authors:** Vivien Hohberg, Reinhard Fuchs, Markus Gerber, David Künzler, Sarah Paganini, Oliver Faude

**Affiliations:** 1 Department of Sports, Exercise and Health, University of Basel, Basel, Switzerland; 2 Department of Sport Psychology, Institute of Sports and Sport Science, University of Freiburg, Freiburg, Germany

**Keywords:** Blended care interventions, Therapist-guided intervention, Digital intervention, Physical activity, Behavior change

## Abstract

**Background:**

Blended care interventions combine therapeutic guidance with digital care. Current research results show the promising role of the blended care approach in clinical care. This new way of delivering health care could have the potential to effectively promote physical activity in different public health settings.

**Objective:**

The aim of the systematic review is to investigate the varieties of intervention characteristics of blended care interventions to promote physical activity in terms of structure, behavior change goals, behavior change techniques, and effectiveness of blended care interventions compared to a control group.

**Methods:**

We searched for randomized controlled trials published from 2000 to March 2021 in MEDLINE, CINAHL, Cochrane Central Register of Controlled Trials, SPORTDiscus, PsycINFO, and Web of Science according to the PRISMA guidelines. Risk of bias was assessed using the Cochrane Collaboration tool. Study characteristics, intervention characteristics, and outcome data were extracted. Furthermore, the effect size on the outcome of physical activity was examined or calculated.

**Results:**

In total, the number of reports identified from the database searches was 4828. Of these, 25 studies were included in the review, with a total of 5923 study participants. Results indicated that the characteristics of blended care interventions showed a high heterogeneity. The combinations of therapist-guided interventions and digital interventions allowed the identification of specific subgroups, but they varied in length (range 8–52 weeks, SD 16.6), intensity, and the combination of the components. The most used combination of blended care interventions to promote physical activity was the combination of one-on-one meetings via telephone and Web-based interventions. Motivational models of behavior change were used most frequently as underlying theoretical foundations. Certain behavior change techniques were used consistently across the individual components, e.g., “problem solving” in the therapist-guided component and “feedback on behavior” in the digital component. Considering the effect size of blended care interventions compared with control groups, most studies showed a small effect.

**Conclusions:**

It can be concluded that blended care interventions have potential to promote physical activity. In the future, further high-quality studies should investigate which type of blended care intervention is effective for which target group. Additionally, insights are required on which intervention characteristics are most effective, taking into account new evidence on behavior change.

*Registration* This systematic literature review was registered in PROSPERO (CRD42020188556).

**Supplementary Information:**

The online version contains supplementary material available at 10.1186/s40798-022-00489-w.

## Key Points

- Blended care interventions have great potential to promote physical activity, regarding their advantages compared to the individual components alone.
- One-on-one meetings via telephone and Web-based interventions were the most frequently used combination of blended care interventions.
- In the context of prevention and rehabilitation, blended care interventions seem to increase physical activity.
- The majority of studies showed small effects of the blended care intervention compared to the control group.

## Background

Physical inactivity is a major risk factor for increased mortality and numerous non-communicable diseases [1]. Worldwide, 7.2% of deaths caused by cardiovascular disease are attributable to physical inactivity. In high-income countries, the prevalence of mortality related to physical inactivity is 9.3% [2]. Physical inactivity also seems to be a major challenge in the COVID-19 pandemic: it is at least as strong as other potentially modifiable risk factors, e.g., smoking, obesity, diabetes, hypertension or cardiovascular disease, for serious disease progression [3]. These current findings highlight an urgent need for action in the area of physical activity promotion. Therefore, in 2018, the “Global Action Plan on Physical Activity 2018–2030” was adopted at the World Health Assembly with the goal of reducing physical inactivity levels by 15% by 2030 [4]. To address the pandemic of inactivity and achieve the goal of the “Global Action Plan,” it is essential to explore new and innovative ways to promote physical activity [5–7].

One way to promote physical activity is to develop and implement lifestyle interventions. Lifestyle interventions in several modes of delivery have the potential to effectively promote physical activity in various target populations [8–11]. Guided by a therapist, lifestyle interventions have multiple benefits, e.g., strengthening social support, establishing high accountability, group dynamic aspects or the possibility to give a direct and tailored feedback. The advantages mentioned help to maintain participants’ intervention adherence [5]. However, studies have shown that 12 months after intervention onset, the initial success in changing the level of physical activity is likely to decrease [12–14]. Hence, a meta-analysis showed only a small effect of therapist-guided interventions 15 months after baseline measurement in terms of increasing physical activity (standardized mean difference, SMD = 0.20) [15]. Furthermore, therapist-guided interventions are expensive, especially if they are to be implemented on population level [16]. Also, types of therapist-guided interventions, e.g., face-to-face interventions, are by nature limited to a specific location depending on where and when the sessions take place [5, 7, 17].

Digitalization and the advent of modern information and communication technologies provide the opportunity to compensate for these disadvantages. Time- and location-independent digital interventions such as smartphone applications (apps) or Web-based interventions to promote favorable health behaviors showed positive impact on behavior change in recent reviews and meta-analyses [18–20]. A major advantage of digital interventions is that they offer broad accessibility. This includes for instance that digital interventions can be used widely across a large number of people, but also further benefits like access to the intervention without waiting time, cost-effectiveness, overcoming stigmatizing barriers through anonymity, and the possibility to provide an intervention to individuals at their own individual pace [21–24]. Web-based interventions alone, though, proved to have only a negligible effect in terms of increasing physical activity in a meta-analysis (SMD = 0.14) [25]. In a further meta-analytic study, an effect of app-based interventions could only be demonstrated in the short term [19, 25]. In addition, adherence of participants to digital interventions is low and dropout rates are high [24, 26, 27]. This may have a negative impact on the expected effect of digital interventions [28]. In addition, digital interventions carry the risk of usability problems, security issues, and privacy concerns [29, 30]. A further meta-analysis showed that app-based interventions to increase physical activity are more effective when they include personal components such as face-to-face sessions, phone calls or text messages from real coaches or therapists [31]. However, it is suggested that interventions that exclusively rely on digital components (“stand-alone” apps or Web-based interventions) are less effective than a combination of digital approaches and additional strategies, such as telephone coaching or traditional face-to-face contact [32, 33].

The term blended care intervention, basically, describes the coordinated combination of therapist-guided interventions and digital interventions. Following Kloek and colleagues [34], we define the two components of blended care interventions to promote physical activity as follows: Therapist-guided interventions are characterized in the broadest sense by the fact that there is an actually existing, personal contact between the therapist, specialist or coach and the participants in the intervention. Examples include individual counseling, group sessions, or sports programs, but also personal contact, which can take place via modern communication channels, e.g., telephone counseling or video-conferencing. In contrast to therapist-guided components, digital interventions are machine-powered. Thus, the digital intervention is automated and there is no personal contact with therapists or professionals. This type of intervention, for example, can be app-based, Web-based, or delivered via automated mails.

Recent research confirms the seminal role of the blended care approach in clinical care [35, 36]. So far, reviews on blended care interventions have focused mainly on the field of psychotherapy [35] or chronic somatic diseases [34]. Current reviews and meta-analyses on physical activity promotion have considered either digital interventions [37] or therapist-guided intervention [14, 38]. The purpose of our review was to provide an overview of the variety of intervention characteristics of blended care interventions. We aimed to answer the following three research questions: (i) How are blended care interventions to promote physical activity structured? (ii) On what theoretical basis are these blended care interventions designed? (iii) What are the effect sizes of the blended care interventions in terms of increasing physical activity compared to the control group? To provide an overview of blended care interventions in the context of physical activity promotion, we examined the structure as well as the components of blended care interventions. In addition, we reviewed the goals of behavior change, behavior change techniques (BCTs), and theories of behavior change. Finally, we conducted an explorative analysis of possible causes of heterogeneous effects of blended care interventions.

## Methods

This systematic review was conducted and reported according to the Preferred Reporting Items for Systematic Reviews and Meta-Analyses (PRISMA) [39] (see Additional file 1). It has been registered a priori in PROSPERO (CRD42020188556). A review protocol was not prepared.

### Search Strategy

The search for eligible studies was conducted in MEDLINE (via PubMed), SPORTDiscus, PsycINFO, Cumulative Index to Nursing and Allied Health Literature CINAHL (all via EBSCO), Web of Science, and Cochrane CENTRAL databases. In each database, the search covered the period from January 2000 to May 19, 2021. The main search was conducted on May 29, 2020, to find studies from January 2000 to May 2020. We updated the results on March 19, 2021 (last search entry), to include additional studies from May 2020 to March 19, 2021. The period was limited from 2000 since there were few studies examining digital interventions to promote physical activity before 2000 and no studies examining blended care interventions. The search term included a combination of the following terms: “physical activity” AND ((“eHealth” AND “face-to-face”) OR “blended intervention”) AND “randomized controlled trial,” and the associated synonyms (see Additional file 2). In each database, a filter was set to include human studies and exclude animal studies. The detailed description of the search strategy for each database can be found in the Appendix. Furthermore, relevant studies were identified via a search of the bibliographies of the included studies, a hand search in Google Scholar, and personal contacts.

### Eligibility Criteria

The criteria for including studies were based on the PICOS scheme (population, intervention, comparator, outcome, study design) [40]. Studies were included if they examined a sample of adults older than 18 years and investigated an intervention to promote physical activity with the outcome of physical activity. For inclusion, the interventions had to comply with the definition of the blended care concept, and hence consist of a digital and therapist-guided component. The intervention had to be based on at least one theory, model or framework as a quality characteristic [41] and had to be compared with a control group (e.g., waiting list, treatment as usual, digital component only, therapist-guided component only). Only randomized controlled trials (RCT) published in English and from 2000 onward were included.

Studies were excluded if the measurement tool used to assess physical activity was not validated. Studies were also excluded if there was no discernible association between digital and therapist-guided components, as for instance, if a therapist-guided intervention is supplemented by a commercial app. The components have to be linked to or based on each other. If the digital intervention consisted of only a form of physical activity tracking (e.g., via pedometers), the study cannot be included in the review, as physical activity tracking alone is not based on a behavioral theoretical concept. Studies are only included if physical activity tracking is part of the theory-based digital or therapist-guided component.

### Study Selection

Duplicates of the studies found were identified, individually reviewed, and removed using a reference program (Citavi 6). VH and DK independently screened studies identified via the different search strategies using the title and abstract of the studies according to the inclusion and exclusion criteria. The results were then compared between the two authors. Discrepancies and disagreements were discussed in order to reach consensus. If the full texts for the studies were not available for screening, the corresponding authors were contacted for access to the full text. The full texts were also screened separately for inclusion by VH and DK. Discrepancies were discussed, and consensus was reached regarding the set of studies to be included.

### Data Extraction

Data were extracted from studies that met the eligibility criteria. We first extracted descriptive data from the included studies. These comprised author(s), publication year, country, comparison group(s), basic sample characteristics, and measurement instrument(s) used to assess physical activity, and measurement time points. To describe the intervention design, we extracted the components of the digital intervention and the therapist-guided intervention. In order to identify the mode of delivery of the digital component, the authors created the following scheme adapted from Webb et al. [42] and Kloek et al. [34]: (1) Web-based, (2) app-based, (3) computer-based, (4) text message (standardized), and (5) automatic phone call. We used the following scheme to extract the therapist-guided component: (1) one-on-one meeting in person, (2) one-on-one meeting via video call, (3) one-on-one meeting via telephone, (4) group session, (5) training, (6) individual text message, and (7) chat. For further description of the intervention, we extracted the design, objectives of behavior change besides the promotion of physical activity, target group, integration of intervention components (parallel vs. sequential), duration of intervention in weeks, and behavior change theory. The integration of intervention components could be divided into parallel, sequential, and parallel–sequential, based on Erbe and colleagues [35]. A parallel sequence of components means that components proceed simultaneously from the start to the end of the intervention. If the components are sequential, the intervention starts with the therapist-guided or digital component and ends with each of the other components. If the components run parallel–sequentially, the therapist-guided component and the digital component start parallel and end with one of the two components.

In addition, we collected data to determine whether there was a group difference in physical activity between intervention and comparison group(s). We further calculated or extracted, whenever possible, the effect sizes and their 95% confidence interval. The BCTs used in the interventions were further recorded using the BCT taxonomy of Michie and colleagues [43]. If the used BCTs were not listed explicitly, they were entered manually. Data extraction was performed independently by VH and DK to avoid errors and outcome bias, especially regarding the identification of BCTs applied in each intervention. If discrepancies were identified in the data extraction, they were discussed with reference to the specific text passages of the studies.

### Quality Assessment

All included studies were assessed for quality using the Risk of Bias Assessment Tool [44]. This tool can be used to assess the risk of bias for randomized controlled trials by inquiring about various potential bias factors. The tool examines several domains that elucidate the randomization process, potential deviations from the planned intervention, missing values, and outcome measurement. Since blinding of participants and study staff to investigate blended care intervention is not possible, the assessment of blinding was omitted according to another systematic review [34]. Disagreements were discussed in order to reach consensus. Finally, a global assessment of the risk of bias was made by VH and DK based on the queried domains.

### Data Analysis

Due to the heterogeneity of the studies and complexity of the interventions, a qualitative evaluation of the narrative synthesis was conducted. The focus of the evaluation was on the composition of the blended care interventions and the analysis and comparison of effect sizes on physical activity together with 95% confidence intervals as an estimate of the uncertainty, and the assessment of methodological quality. To calculate the effect size, Cohen's d was used. Cohen's d was either taken from the studies if the value was reported or calculated using the formula $d=\frac{{\overline{x} }_{1}- {\overline{x} }_{2}}{s}$, with ${\overline{x} }_{1}$ and ${\overline{x} }_{2}$ being the mean outcome values of the control group and the intervention group. Pooled standard deviation was used when groups were unequally sized (${\mathrm{SD}}_{\mathrm{pooled}=}\sqrt{\frac{\left({n}_{1}-1\right)\times {s}_{1}^{2}+\left({n}_{2}-1\right)\times {s}_{2}^{2}}{\left({n}_{1}-1\right)+ \left({n}_{2}-1\right)}}$, with ${n}_{1}$ and ${n}_{2}$ being the group size of the intervention group and the control group and ${s}_{1}$ and ${s}_{2}$ the standard deviation of the particular groups). The endpoint of the intervention was chosen to calculate the effect size on physical activity. When possible, effect sizes were reported with a 95% confidence interval. According to the guidelines of Cohen [45], values of < 0 correspond to a negative effect, < 0.2 to a negligible effect, 0.2 to 0.4 to a small effect, 0.5 to 0.7 to a medium effect, and ≥ 0.8 to a large effect. By means of the assessment of study quality, we weighted the studies with respect to their robustness. We used extracted data to analyze the intervention design. Finally, we provided an overview of the intervention structure of blended care interventions and an interpretation for possible causes of heterogeneous effects.

The results of the search are illustrated in the PRISMA flowchart. An overview of the studies is presented via a table. In addition, the combinations of blended care interventions, behavior change goals, and BCTs used are presented graphically for overview.

## Results

### Study Selection

**Fig. 1:**
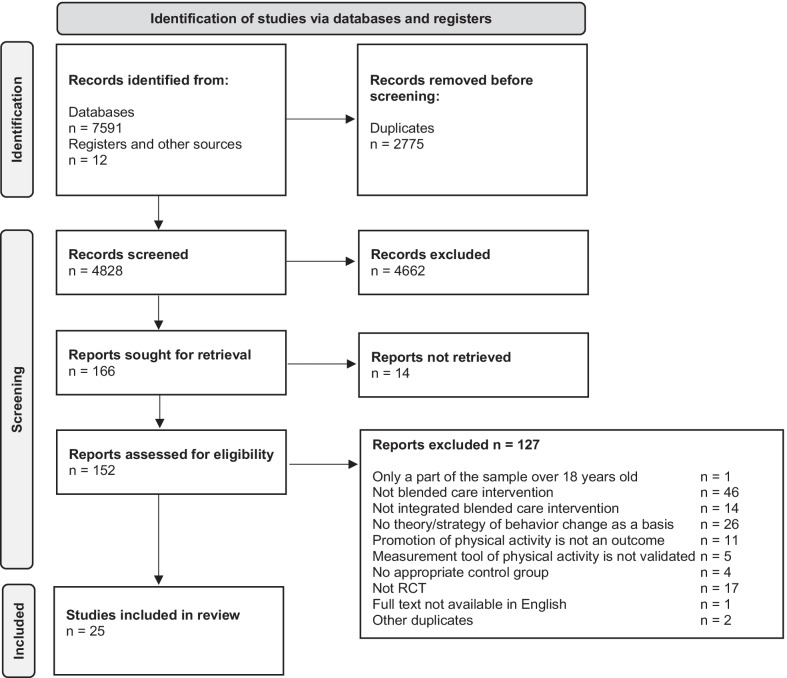
PRISMA flowchart of included and excluded studies [39]

The PRISMA flowchart (Fig. 1) provides an overview of the study search and selection process. The literature search of the databases resulted in 7591 findings, and the manual search yielded 12 results. After removal of duplicates, 4828 studies remained, which were screened for title and abstract. A total of 152 studies were eligible for full-text screening, of which 25 were included in the review.

### Design of the Studies

Table 1 gives an overview of the study characteristics. The total number of included participants in all RCTs was 5923 with a range of 64 to 637 participants per study. In total, 61.3% of the participants were female. The mean age of the participants across all studies was 49 years, ranging from 31.1 to 70.2 years. The most commonly used method to measure physical activity was via questionnaire (68%, 17/25) [46–62]. Of these, the International Physical Activity Questionnaire-Short Form (17.6%; 3/17) was used most frequently [54–56]. Furthermore, 20% (5/25) of the studies used accelerometers to measure three-dimensional acceleration [51, 63–66] and 16% (4/25) used pedometers to count steps [62, 67–69]. The measurement time points of the outcome (physical activity) ranged from 0 (baseline) to 12 months. In total, 28% of the studies measured physical activity after the end of the intervention during follow-up.

**Table 1:** Summary of blended care intervention studies

References	Blended care intervention (IG)	Control groups (CG)	Baseline n (IG)	Baseline n (CG)	Mean age (SD)	Female (%)	Measurement method	Duration (week)	Delivery mode	Target group	Theory/strategies of behavior change	Cohen’s d (95% confidence interval)	Risk of bias
Albright et al. USA [46]	Individual meeting, Web-based intervention	Digital intervention	154	157	31.9	100	MVPA min/week (questionnaire)	52	Parallel	Postpartum women	Motivational interviewing	+ 0.36 ^(1)^	Low
Alley et al. Australia [47]	Individual meeting, Web-based intervention	Digital intervention, waiting list	126	80	54	76	PA min/week (questionnaire)	8	Parallel	Inactive adults	Theory of Planned Behavior, Elaboration Likelihood Model	+ 0.55 ^(1) (2)^	Some concerns
Anderson et al. UK [63]	Individual meeting, Web-based intervention	Treatment as usual	39	39	47.1 (12.8)	88	Change of moderate PA min/day (accelerometer)	12	Parallel	Adults with cancer screening	Social Cognitive Theory, Self-Regulating Theory, Health Action Process Approach	+ 0.25 (−0.33; 0.83)	Some concerns
Broekhuizen et al. Netherlands [48]	Individual meeting, Web-based intervention	Treatment as usual	181	159	45.3	57	MVPA min/week (questionnaire)	52	Sequential	Adults with familial hypercholesterolemia	Integrated Model for Exploring Motivational and Behavioral Change, motivational interviewing	-^(3)^	Low
Christian et al. USA [49]	Individual meeting, computer-based intervention	Treatment as usual	155	155	53.2	66	Change in PA MET min/week (questionnaire)	40	Sequential	Overweight adults with diabetes	Motivational interviewing	+ 0.59 (0.35; 0.83)	Some concerns
Collins et al. USA [67]	Individual meeting, text messages, app-based intervention	Treatment as usual	35	34	58.7 (6.8)	86	Steps min/week (pedometer)	26	Parallel	Latinos > 50 years	Motivational interviewing, patient-centered assessment and counseling for exercise	+ 0.34 (−0.14; 0.82)	Low
Crane et al. USA [50]	Individual meeting, Web-based intervention	Waiting list	53	54	44.2	0	PA in caloric expenditure kcal (questionnaire)	24	Sequential	Overweight/ obese men	Self-Determination Theory, Social Cognitive Theory	–^(4)^	Low
Duncan et al. Australia [51]	Individual mail, text messages, app-based intervention	Waiting list	80	36	44.5 (10.4)	71	MVPA min/week (accelerometer), MVPA min/day (questionnaire)	52	Parallel	Adults with BMI > 25	Social Cognitive Theory, Self-Regulating Theory	–^(4)^	Low
Fischer et al. Switzerland [52]	Individual meeting, text messages, Web-based intervention	Digital intervention	93	96	42.2 (11.4)	68	MVPA min/week (questionnaire)	26	Parallel	Inactive adults	Motivation and Volition Theory, Behavior Change Wheel	+ 0.33 ^(1) (5) (6)^	Some concerns
Glasgow et al. USA [53]	Individual meeting, group sessions, Web-based intervention, automatic phone call	Digital intervention, treatment as usual	331	132	58.4 (9.2)	50	PA in caloric expenditure per week (questionnaire)	16	Parallel	Adults with diabetes type 2	Social Cognitive Theory, Self-Efficacy Theory, “5 As” Self-Management Model	+ 0.23 ^(1) (7)^	Some concerns
McDermott et al. USA [64]	Individual meeting, group sessions, Web-based intervention	Treatment as usual	99	101	70.2	53	PA min/day (accelerometer)	40	Parallel	Adults with peripheral artery disease	Social Cognitive Theory	−0.01 (−0.3; 0.25)	Some concerns
Morgan et al. Australia [68]	Individual meeting, individual mail, Web-based intervention	Treatment as usual	34	31	35.9 (11.1)	0	PA in steps min/week (pedometer)	12	Parallel	Overweight, obese adults	Social Cognitive Theory	–^(4)^	Low
Mouton and Cloes Belgium [54]	Training, group session, Web-based intervention	Digital intervention, therapist-guided intervention, waiting list	52	52 (DI) 52 (TG) 50 (WL)	65.3	64	PA in MET min/week (questionnaire)	12	Parallel	Adults > 50 years	Transtheoretical Model, Stages of Change Model	+ 0.2 ^(1) (2)^	Some concerns
Partridge et al. Australia [55]	Individual meeting, individual mail, text messages, app- and Web-based intervention	Digital intervention	123	125	27.4	61	PA in MET min/week (questionnaire)	12	Parallel	Young adults at risk of weight gain	Transtheoretical Model, Stages of Change Model	+ 0.16 (−0.09; 0.41)	Low
Plotnikoff et al. Australia [69]	Group session, training, app-based intervention	Waiting list	42	42	44.7 (14.0)	70	Steps min/week (pedometer)	20	Parallel, sequential	Adults with diabetes type 2	Social Cognitive Theory, Health Action Process Approach	+ 0.56 ^(1)^	Low
Richardson et al. USA [70]	Chat, individual mail, Web-based intervention	Digital intervention	254	70	52 (11.4)	65	Steps min/day (pedometer)	16	Parallel	Adults with BMI > 25, diabetes type 2, coronary artery disease	Social Cognitive Theory, Social Learning Theory	+ 0.38 (0.11; 0.64)	Some concerns
Rubinstein et al. Argentina [56]	Individual meeting, text messages	Treatment as usual	316	321	43.4	54	PA in MET min/week (questionnaire)	52	Parallel	Adults with prehypertension	Transtheoretical Model, Health Belief Model	–^(3)^	Low
Schaller et al. Germany [57]	Individual meeting, group session, chat, Web-based intervention	Treatment as usual	201	211	50.4	31	PA in MET min/week (questionnaire)	29	Parallel, sequential	Adults with orthopedic disorders	Motivation and Volition Theory, Rubicon Model of Action Phases	+ 0.09 (−0.10; 0.28)	Low
Sniehotta et al. UK [65]	Individual meeting, individual mail, text messages, Web-based intervention	Digital intervention	144	144	41.8	77	PA min/day (accelerometer)	52	Parallel	Adults with previous weight loss	Self-Regulating Theory, Health Action Process Approach	+ 0.12 (−0.12; 0.37)	Low
Steele et al. Australia [62]	Individual meeting, Web-based intervention	Digital intervention, therapist-guided intervention	65	62 (DI) 65 (TG)	38.7 (12.0)	83	MVPA min/week (questionnaire) Steps min/day (pedometer)	12	Parallel	Inactive adults	Social Cognitive Theory	TG: −0.21 (−0.56; 0.13) DI: −0.31 (−0.66; 0.04)	Low
Torbjørnsen et al. Norway [71]	Individual meeting, chat, app-based intervention	Digital intervention, treatment as usual	50	51 (DI) 50 (TAU)	57	41	Change in PA (questionnaire)	52	Parallel, sequential	Adults with diabetes type 2	Motivational interviewing, Transtheoretical Model, Problem-Solving Model	–^(4)^	Some concerns
Turner et al. USA [59]	Individual meeting, computer-based intervention	Treatment as usual	31	33	53.1	36	PA in MET min/week (questionnaire)	26	Parallel	Adults with multiple sclerosis	Motivational interviewing	+ 0.92 (0.40; 1.44)	Some concerns
van der Weegen et al. Netherlands [66]	Individual meeting, individual mail, app- and Web-based intervention	Treatment as usual, therapist-guided intervention	65	68 (TAU) 66 (TG)	57.9	51	MVPA in MET min/week (accelerometer)	26	Parallel	Adults with chronic obstructive pulmonary, diabetes type 2	“5 As” Self-Management Model	+ 0.3 ^(1)^	Low
Wilbur et al. USA [60]	Group session, automatic phone call	Therapist-guided intervention	97	95	53.1 (6.5)	100	MVPA min/week (questionnaire)	48	Parallel	Sedentary African-American women	Social Cognitive Theory, Motivational interviewing	+ 0.21 (−0.09; 0.51)	Low
Wylie-Rosett et al. USA [61]	Individual meeting, group session, computer-based intervention	Treatment as usual	236	236	52.2	82	PA in walking min/day (questionnaire)	52	Parallel	Adults with BMI > 25	Transtheoretical Model	+ 0.32 (0.09; 0.54)	Some concerns

*IG* intervention group, *CG* control group, *PA* physical activity, *MVPA* moderate-to-vigorous physical activity, *MET* metabolic equivalent, *BMI* body mass index, *DI* digital intervention, *WL* waiting list, light: blended care intervention light, *TG* therapist-guided intervention, *TAU* treatment as usual

^(1)^Calculation of the confidence interval not possible, since no standard deviation (SD) was specified. ^(2)^ Compared to waiting list. ^(3)^ Geometric means was used. ^(4)^ Calculation of the effect size not possible, since no SD was specified. ^(5)^ Compared to the digital intervention. ^(6)^ Effect sizes calculated from group with digital intervention and group with blended care intervention (no values given for blended care intervention group alone). ^(7)^ Compared to treatment as usual

### Target Groups

The blended care interventions addressed different target groups. Almost half of the interventions examined targeted a group with a medical condition (48%; 12/25) [48, 49, 53, 56, 57, 59, 63, 64, 66, 69–71], and of these, six interventions (50%) targeted people with type 2 diabetes [49, 53, 66, 69–71]. Overweight people with and without a preexisting medical condition were addressed by 24% (6/25) of the interventions examined [49–51, 61, 68, 70], and 16% (4/25) targeted inactive people or people who engage in sedentary behaviors [47, 52, 59, 60, 62]. In total, 8% (2/25) of the interventions focused specifically on older adults over 50 years [54, 67].

### Intervention Duration and Type of Integration

Blended care interventions varied in duration. The mean duration of the interventions was approximately 30 weeks (range 8–52 weeks, SD 16.6). In the majority of interventions, the therapist-guided and digital components took place in parallel (76%; 18/25) [46, 47, 52–56, 59–68, 70]; in 3 of 25 interventions (12%), the therapist-guided and digital components were sequentially linked [48–50]; and in 4 of 25 interventions (15%), the therapist-guided and digital components took place at least partially simultaneously (parallel–sequential) [51, 57, 69, 71].

### Intervention Components

**Fig. 2:**
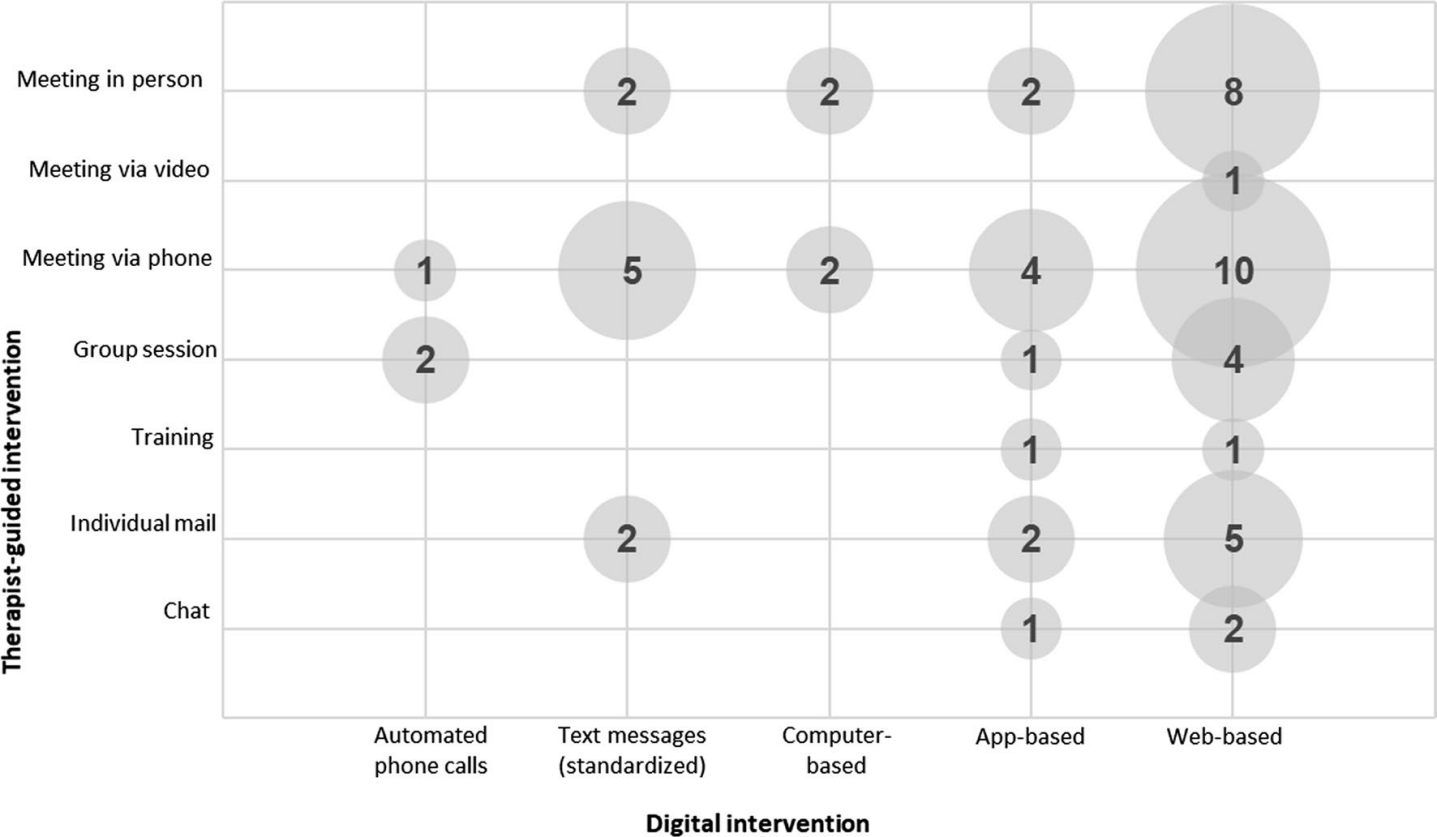
Frequency of intervention components of blended care interventions

Figure 2 represents how often the individual therapist-guided and digital components were combined in the interventions. The size of the bubble represents how often a specific combination was used. When observing and interpreting the data, it is important to note that a single intervention may include several of the components and combine more than two.

With 40% (10/25), the most commonly used blended care intervention was the combination of one-on-one meetings via telephone and Web-based interventions [46, 48, 52, 53, 55, 57, 63–66], followed by one-on-one meetings in person and Web-based interventions (32%; 8/25) [48, 50, 62–66, 68], individualized mails and Web-based interventions (20%; 5/25) [55, 65, 66, 68, 70], one-on-one meetings via telephone and text messages (20%; 5/25) [52, 55, 56, 65, 67], and one-on-one meetings via telephone and app-based interventions (16%; 4/25) [55, 66, 67, 71].

Considering the individual blended care components, the most commonly used therapist-guided components were one-on-one interviews via telephone (60%; 15/25) [46, 48, 52, 53, 55–57, 59, 61, 63–67, 71], one-on-one in-person interviews (40%; 10/25) [48–50, 61–66, 68], and group sessions (28%; 7/25) [53, 54, 57, 60, 61, 64, 69]. The focus of digital components was on Web-based interventions (64%; 16/25) [46–48, 50, 52–55, 57, 62–66, 68, 70], app-based interventions (24%; 6/25) [51, 55, 66, 67, 69, 71], and/or standardized text messaging (24%; 6/25) [51, 52, 55, 56, 65, 67].

### Behavioral Goals

**Fig. 3:**
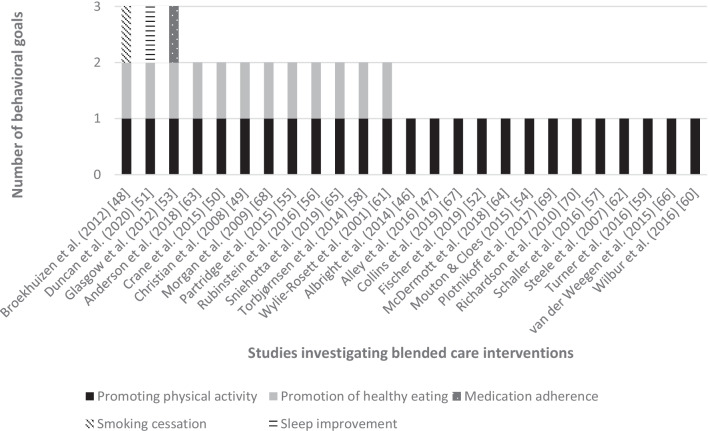
Number of promoted behavioral goals in blended care interventions

Based on the defined inclusion criteria, all of the investigated blended care interventions had the goal of promoting physical activity. In addition to this goal, four other behavior change goals were addressed: Healthy eating (48%; 12/25) [49–51, 53, 55, 56, 61, 63, 65, 68, 71, 72], medication adherence (4%; 1/25) [53], smoking cessation (4%; 1/25) [48], and sleep improvement (4%; 1/25) [51] (see Fig. 3). Of the 25 blended care interventions reviewed, 48% (12/25) examined multiple behavioral goals. Three interventions (25%) addressed a total of three behavioral goals [48, 51, 53, 71] and eight interventions (66%) addressed two behavioral goals [49, 50, 55, 56, 61, 63, 65, 68]. All other 13 interventions (52%) targeted a unimodal goal, meaning that they exclusively aimed at promoting physical activity [46, 47, 52, 54, 57, 59, 60, 62, 64, 66, 67, 69, 70].

### Theoretical Basis

All blended care interventions were based on a theoretical model of behavior change according to the established inclusion criteria. The theory-based approach to behavior change is a quality characteristic of behavior change interventions, as these interventions have been shown to be effective in terms of behavior change [41]. The theories and models of behavior change used in the blended care interventions could be divided into four categories: Cognitive motivational models of health behavior, stage and process models of behavior change, practice-oriented models or frameworks, and ecological models. Of all blended care interventions reviewed, 64% (16/25) had more than one theory as a foundation, with a total number of 18 different theories in all 25 interventions identified. Of the theories integrated in the blended care interventions, 64% (16/25) could be assigned to cognitive motivational models of health behavior [47, 50, 51, 53, 56, 60, 62–65, 68–70]. Here, the social cognitive theory [73] was most frequently used as a theoretical basis (63%; 10/16) [50, 51, 53, 60, 62–64, 68–70]. The stage and process models of behavior change included 14 cited theories [48, 52, 54–57, 61, 63, 65, 69, 71], with the transtheoretical model [74] (36%; 5/14) representing the most frequent basis for blended care interventions in this category [54–56, 61, 71]. Among the ten practice-oriented models and frameworks mentioned [46–49, 52, 53, 59, 66, 67, 71], motivational interviewing [75] was used most frequently (60%; 6/10) [46, 48, 49, 59, 67, 71]. Two theories, social ecological model [76] and social learning theory [77], were assigned to the ecological models, with one mention each [53, 70].

### Behavior Change Techniques

In total, 42 (45%) of 93 BCTs included in the BCT taxonomy [43] were used at least once across all studies. The reviewed interventions used in total an average of 11.3 BCTs (range 5–22). Of these, 6.2 BCTs (range 1–13) were used on average for the therapist-guided intervention and 5.3 BCTs (range 1–10) were used for the digital intervention. BCTs from 4 of 16 superordinate categories of the BCT taxonomy were used most frequently. These were *goals and planning*, *feedback and monitoring*, *social support*, and *natural consequences*. Considering the blended care intervention in general, the most frequently used BCTs in the interventions were *problem solving* (96%; 24/25) [46–57, 59–67, 69–71], followed by *goal setting* (behavior) (88%; 22/25) [46, 47, 50–57, 59–69, 71], *feedback on behavior* (76%; 19/25) [46–57, 59, 60, 63, 65, 66, 68, 69], *self-monitoring of behavior* (76%; 19/25) [46, 47, 50–55, 57, 59, 60, 62–66, 68, 69, 71], and *social support (unspecified)* (68%; 17/25) [46, 48, 49, 52–55, 57, 59, 60, 62–64, 67–69, 71].

**Fig. 4:**
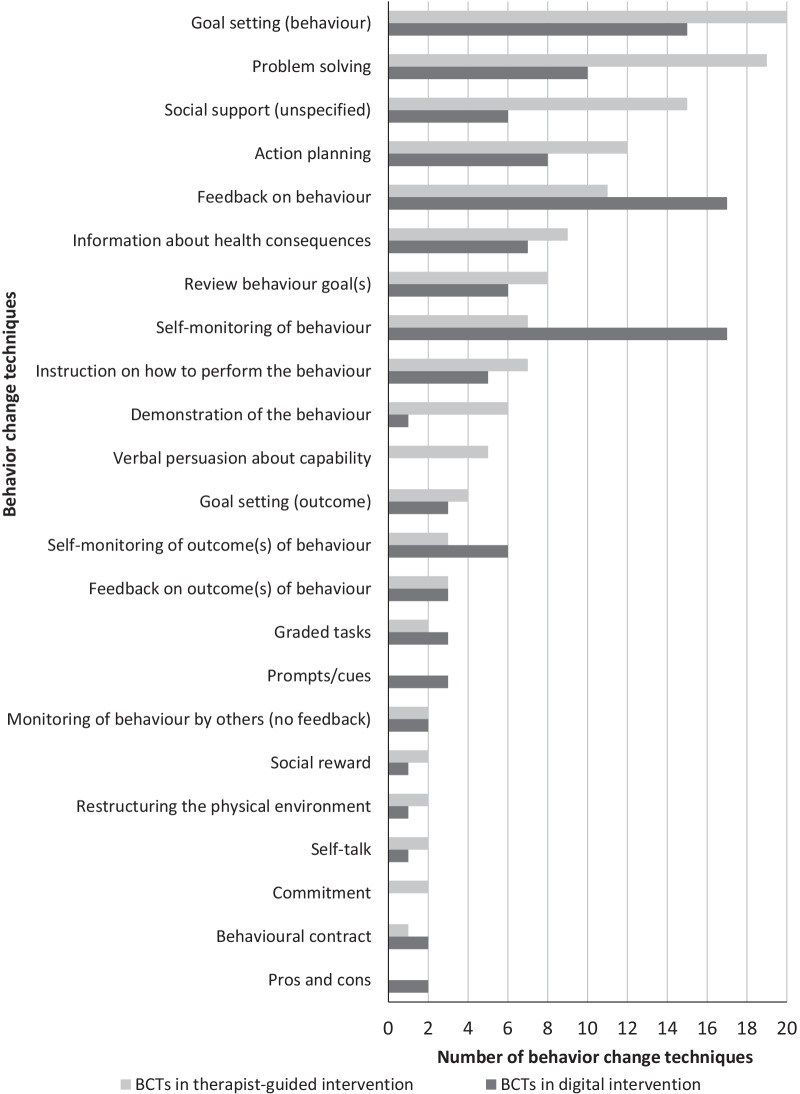
Number of behavior change techniques (BCTs) in therapist-guided and digital components of blended care interventions

Figure 4 shows a comparison of the BCTs used in the therapist-guided and digital components. For the sake of clarity, only BCTs that were used at least twice in the particular component across all interventions are shown. Comparing the distribution of BCTs in the individual components, it became noticeable that most BCTs were used in both components. Only *verbal persuasion about capability* (100%; 5/5) [46, 47, 49, 53, 69] and *commitment* (100%; 2/2) [53, 63] were used exclusively in the therapist-guided component and *prompts/cues* (100%; 3/3) [51, 59, 67] and *pros and cons* (100%; 2/2) [55, 57] in the digital component. In comparison, however, the use of these mentioned BCTs was low in terms of frequency. There were other BCTs that were used more frequently than others in the particular components. A BCT can be used in one of the two intervention components or in both. Thus, the frequency data of the BCTs refer to the use of the particular BCT in both components. *Problem solving* (65.5%; 19/29) [46, 52, 53, 55–57, 59–67, 69–71], *social support (unspecified)* (71.4%; 15/21) [46, 48, 49, 52–54, 59, 60, 62–64, 67–69, 71], and *demonstration of the behavior* (85.7%; 6/7) [54, 59–61, 63, 64, 69] were used particularly frequently in the therapist-guided component, and *feedback on behavior* (60.7%; 17/28) [47–57, 59, 60, 65, 66, 68, 69], *self-monitoring of behavior* (70.8%; 17/24) [46, 47, 51–55, 59, 60, 62–66, 68, 69, 71], and *self-monitoring of outcome(s) of behavior* (66.7%; 6/9) [51, 52, 55, 65, 70, 71] in the digital component.

### Effect Sizes and Study Quality

A total of six studies reported neither effect size nor the values needed to calculate effect size [48, 50, 51, 56, 68, 71]. The range of effect sizes of the other 19 studies investigating blended care interventions was between −0.31 and + 0.92, i.e., from a negative (detrimental) to a large positive (beneficial) effect. Overall, 56% (14/25) of the studies had a low risk of bias, 44% (11/25) had some concerns, and no study was rated with a high risk of bias (see Table 1). Here, most of the concerns were about outcome measurement (see Additional file 3). Of the 25 reviewed blended care interventions, two interventions (8%) showed a negative effect size [62, 64]. One of these blended care interventions [64] was tested in comparison with treatment as usual (TAU) with some concerns about risk of bias, and one [62] was compared to digital intervention group and therapist-guided intervention group with a low risk of bias. Of the 25 blended care interventions, three interventions (12%) showed no relevant effect (*d* = 0.00 to + 0.19) compared with TAU [57] or the digital intervention alone [55, 65]. In terms of effect size, most studies (40%; 10/25) revealed a small effect (*d* =  + 0.20 to + 0.49) [46, 52–54, 60, 61, 63, 66, 67, 70]. In total, 40% (4/10) of the studies with a small intervention effect had a low risk of bias [46, 55, 57, 60, 65–67], and all other studies indicated some concerns regarding the risk of bias (60%; 6/10) [52–54, 63, 70]. Four of the 25 blended care interventions (16%) demonstrated medium (*d* =  + 0.50 to + 0.79) to large effects (d ≥  + 0.80) with a range from + 0.55 to + 0.92 [47, 49, 59, 69]. Thereof, three of four studies were rated with some concerns of risk of bias [47, 49, 59] and one study had a low risk of bias [69]. The effects of the blended care interventions with a medium-to-large effect referred to a control group that either received TAU [49, 59] or was on the waiting list [47, 69].

Comparing studies that used objective physical activity measurement methods with those that used self-reported physical activity methods, no pivotal difference is obvious regarding effect size. Of the studies that showed a small effect size (*d* =  + 0.20 to + 0.49) of the blended care intervention in terms of physical activity, 44.4% (4/9) [63, 66, 67, 70] used an objective method and 35.3% (6/17)[46, 52–54, 60, 61] used a self-reported method to measure physical activity. Blended care interventions that demonstrated a medium effect size (*d* =  + 0.50 to + 0.79) on physical activity 11.1% (1/9) [69] used objective methods and 11.8% (2/17) [47, 49] used self-reported measurement methods. Studies demonstrating negative (d ≤ 0.00), negligible (*d* = 0.00 to + 0.19), or large effect sizes (*d* ≥  + 0.80) on physical activity also indicated no discernible differences between measurement methods.

## Discussion

This systematic literature review provides an overview of blended care interventions designed to promote physical activity. We evaluated blended care interventions with regard to type of integration, intervention duration, intervention components, target groups, intervention goals, behavior change techniques (BCT), and theories of behavior change, as well as the effects of the investigated blended care interventions.

### Key Results

Parallel integration of the digital and therapist-guided components dominated as the type of integration. The most used combination referring to the components of blended care interventions was the combination of one-on-one meetings via telephone and Web-based interventions. With regard to the individual components of the blended care interventions, one-on-one interviews via telephone were also used most frequently in the therapist-guided component and Web-based interventions in the digital component. The addressed target groups of blended care interventions were generally different, but addressed primary and secondary prevention almost equally. Looking at the behavior change goals of the studied blended care interventions, just under half aimed at promoting physical activity and included the promotion of healthy eating as an additional goal. Motivational theories/models of health behavior were the most commonly used with social cognitive theory the most frequently used model/theory in the blended care interventions. Considering the smallest units of behavior change, some BCTs were used more frequently in the individual components, e.g., *problem solving* in the therapist-guided component and *feedback on behavior* in the digital component. Regarding the effect sizes of blended care interventions compared to control groups, most studies showed a small effect.

#### Intervention Duration and Type of Integration

With regard to the duration of the blended care interventions, a large range (from 8 to 52 weeks) was observed. This heterogeneity in length of interventions is in line with a review that examined blended care interventions for behavior change in people with chronic somatic disorders (range 5–52 weeks) [34]. In addition, there was evidence that adherence to online interventions is higher when the interventions are shorter [78]. However, the necessity of long-term implementation of behavior change needs to be considered [79]. Digital interventions as booster treatments could improve the effects of therapist-guided interventions to promote behavior change [36, 80]. Although the use of parallel integration of the therapist-guided and digital components was prevalent, we found no far-reaching differences between the modes of delivery in terms of effect size in our review. James and colleagues [81] reported that both parallel and sequential approaches were described in the studies as more effective than the specific control intervention. However, there is no evidence that a parallel mode of delivery is more effective than a sequential mode of delivery [81].

#### Intervention Components

One-on-one meetings via telephone were most frequently used as a therapist-guided component in the blended care interventions, likely due to the fact that personal contact with the therapist, expert, or coach was given, but barriers that existed in one-on-one in-person interviews or group sessions were omitted. These include for example the dependence on location. Although one-on-one interviews via telephone represented the therapist-guided component that was used the most, over half of the blended care interventions had an integrated in-person component (one-on-one in-person interviews, group sessions, training). A review by Carrillo de Albornoz and colleagues [82] stated that therapist-guided interventions delivered in-person and therapist-guided interventions delivered via distance showed no difference in terms of effectiveness in health-related outcomes. Future studies should examine whether and for which target group an in-person component, a component via distance or a mixed mode of delivery, is more effective in blended care interventions targeting the promotion of physical activity.

Our results showed that Web-based interventions were the most commonly implemented digital component in blended care interventions. This kind of digital intervention can be highly engaging to patients from the perspective of convenience, ease of access, and the ability to maintain anonymity or privacy [83]. While systematic reviews have found positive effects on health-related outcomes, effect sizes are generally small or negligible [25, 83, 84]. This review showed, when Web-based interventions were combined with a therapist-guided component, comparable effect sizes were reported. In recent years, app-based components in blended care interventions have been explored increasingly. In addition, the app-based component was also commonly used as the digital component in blended care interventions. This may be associated with the use of mobile devices and its related advantages. Over 80% of the population in Europe use smartphones in their daily life [85]. In contrast to Web-based interventions, app-based interventions have the advantage of being available to users constantly and regardless of location [32, 86]. Thus, further implementation and research of app-based interventions in blended care interventions should be encouraged.

#### Target Groups

Both preventive and rehabilitative contexts to promote physical activity should be used to inhibit (further) consequences of inactivity [1]. The results of this review indicate that blended care interventions to promote physical activity are used about equally frequently for target groups in prevention or in rehabilitation. The reported effect sizes do not suggest that blended care interventions may be more effective for either prevention or rehabilitation. Vulnerable groups like older adults benefit from interventions to promote physical activity, as well [87]. Furthermore, digital interventions with the possibility of contacting healthcare providers are associated with higher adherence among vulnerable groups [88]. Currently, no blended care interventions have been designed specifically for seniors. However, as there is evidence that seniors may benefit from blended care interventions to promote physical activity, blended care interventions should be designed for and evaluated in this specific target group. In addition to seniors, it is also reasonable to address children and adolescents as a target group of blended care interventions. The inclusion of children and adolescents in the digital transformation is required in order to establish strong health and well-being at a young age [89]. The prevalence of digital technologies in this target group is high. Approximately 71% of children and young adults aged 15–24 worldwide are online [90]. This open attitude toward digital technologies, in combination with expert guidance, could provide promising results in terms of children and adolescents' health behaviors.

#### Behavior Change Goals, Theoretical Basis, and Behavior Change Techniques

Just under half of the studied blended care interventions had the goal of promoting healthy eating in addition to promoting physical activity. Especially in diseases such as type 2 diabetes, establishing a healthy diet as a behavior change in addition to promoting physical activity is an evident strategy to prevent the progression of the disease [91, 92].

The majority of the used theories of behavior change could be assigned to the category cognitive motivational models of health behavior. The previous public health approach is also based on the strategies of cognitive and motivational theories. These include improving awareness and promoting knowledge, belief, and outcome expectation [93]. However, this approach did not show promising results in promoting physical activity [94]. Cognitive motivational models of health behavior assume that people in general are able to make rational decisions and critically review decisions for or against a particular health behavior. Affective processes, which are intuitive or impulsive, are not considered [93, 95]. As a complement to the theories currently being used, the implementation of theories such as the affective–reflective theory [96], which takes the role of momentary and anticipated affect into account, should be implemented and evaluated in further blended care interventions to promote physical activity.

Based on the applied theories or frameworks of behavior change, no explicit assumptions can be made regarding the effect size. It is worth mentioning here that two of six (33%) studies that used motivational interviewing showed a large effect size [49, 59]. Nevertheless, the two studies showed some concerns regarding the risk of bias. Frost and colleagues [97] confirm that more high-quality research on motivational interviewing is needed.

The results of implemented BCTs could indicate that the implementation and realization of some BCTs are more suitable for the specific components than others. Self-monitoring in particular can be implemented successfully using digital interventions, since behavior or behavioral outcomes can be entered directly in digital form or tracked using devices such as pedometers or accelerometers [98, 99]. In contrast, problem solving, which requires a high level of reflection, can probably be better implemented in the therapist-guided component, because the therapist or coach can support implementation and reflection [100]. Social support, through contact with the therapist or coach or through a peer group, is more appropriate in the therapist-guided intervention as well and has the potential to improve health-related outcomes [101]. Thus, future investigations should examine not only the short- and long-term effectiveness of BCTs [102], but also which BCTs are more appropriate in each component of blended care interventions to promote physical activity.

#### Size of Intervention Effects

The majority of studies showed small effects of the blended care intervention on physical activity compared to the control group. This is consistent with the results of meta-analyses that examined lifestyle interventions to promote physical activity (*d* = 0.26) [103]. Other reviews that have examined blended care interventions in different target groups and outcomes show inconsistent results in terms of effect size: In a meta-analysis of patients with chronic obstructive pulmonary disease, blended care interventions to promote self-management show positive effects on exercise capacity, quality of life, and admission rate [104]. For behavior change in patients with chronic somatic disease, blended care interventions showed inconsistent evidence for most of the studied behavioral outcomes, including physical activity [34]. Erbe and colleagues [35] concluded that blended care interventions for mental illness can be feasible and effective. This review showed promising results of blended care interventions for physical activity promotion with regard to the described effect sizes. However, the investigated studies do not provide evidence that certain characteristics of blended care interventions may be more effective than others. Three of the four studies indicating moderate-to-large effect sizes used one-on-one interviews as a therapist-guided component. As three of these studies showed some concerns regarding the risk of bias, it remains speculative whether blended care interventions with a one-on-one interview as a therapist-guided component are more effective than others. Future research needs further high-quality studies that examine the effectiveness of blended care interventions in promoting physical activity with consideration of the previously discussed characteristics of blended care interventions.

Comparing the blended care intervention to the digital components, most blended care interventions show a small but beneficial effect. This allows conclusions about the relevance of therapist-guided components: It can be assumed that an intervention effect could be achieved by adding the therapist-guided component and its benefits. However, this needs to be verified by statistical analysis for specific target groups and in regard to the individual components. Since only two studies compared the blended care intervention with a therapist-guided component, it is difficult to draw conclusions here. All studies showing medium-to-large effects were comparing blended care intervention with TAU or a waiting list group. But the majority of studies that compared blended care intervention with TAU, however, showed a small effect size. Nevertheless, blended care interventions appear to be an effective and useful adjunct to TAU for promoting physical activity.

### Strengths and Limitations

The review contains some strengths that are worth mentioning. To avoid effect estimates indicating extreme benefits of effects on health-related outcomes, only randomized controlled trials were included. To assess the influence of bias on the study results, the Cochrane Risk of Bias Assessment Tool was used. The tool is based on a domain-based approach and empirical evidence to assess the risk of bias and is therefore characterized by high quality [105]. Overall, the quality of the studies can be rated as good. There were no studies rated with a high risk of bias, but over half of the studies with a low risk of bias. The review provides a detailed overview of the structure and foundation of blended care interventions to promote physical activity and its effect size.

Some limitations of our review need to be considered. The definition of blended care interventions was treated differently in the literature. Particularly in the area of mental health, blended care interventions are defined by a face-to-face component in terms of a one-on-one session or a group setting and a Web-based intervention [106, 107]. In this review, the face-to-face component was expanded to include all possibilities of personal contact, which can also occur via modern communication tools. Furthermore, while we surveyed the outcome physical activity in this review, there was a lack of consistency in the measurement of physical activity across the studies. To compensate for this limitation, we only included studies with a validated instrument to measure physical activity. Another limitation relates to the lack of description of the intervention, particularly of the applied BCTs and how they were implemented in the interventions. In extracting the BCTs, we could only refer to what was described in the studies and, if applicable, in the associated study protocols. Accordingly, if BCTs were insufficiently described, it is possible that more BCTs were used in the blended care interventions than listed in this review. Although the taxonomy of BCTs by Michie and colleagues [43] is available, the terminologies of BCTs have not been used consistently. Under the four-eyes principle, BCTs were extracted and discussed if the listed BCTs were not coded according to Michie and colleagues [43]. The identification of BCTs in the studies using the consistent terminology has already been noted as a limitation in other reviews [108]. Thus, there is a need to ensure that the BCTs are described consistently in a standardized way and that sufficient information about the intervention components is available.

Results indicated that the characteristics of blended care interventions show a high heterogeneity. Due to the heterogeneity of the studies and complexity of the interventions, no meta-analyses could be performed. The investigated blended care interventions allowed the identification of specific subgroups of therapist-guided and digital components, but they vary in length, intensity, and the number of combinations. Thus, currently, it appears difficult to draw far-reaching conclusions about possible effects for specific target groups to promote physical activity. Subsequent studies should investigate which combinations are particularly suitable for which target group. Moreover, the comparison of the specific intervention types with each other should be examined more thoroughly.

## Conclusion and Outlook

Blended care interventions offer many possible combinations of the therapist-guided component and the digital component. The investigated blended care interventions vary widely in their characteristics regarding mode of delivery, length, embedding dosage of individual components, their theoretical basis, and use of BCTs. There is evidence that blended care interventions have a positive, but small beneficial effect on promoting physical activity. However, this should be verified in a more detailed quantitative analysis of particular combinations of the therapist-guided and digital components, as soon as a sufficient number of appropriate studies are available in future. The findings of this review not only provide conclusions for existing blended care interventions to promote physical activity, but can also offer guidance for the design of future blended care interventions, e.g., to use specific BCTs in the individual intervention components. When designing future interventions that include a digital and therapist-guided component, the discussed results of this review regarding the benefits of blended care interventions, type of integration, choice of component, target group, and behavioral goals, theory-based, and BCTs should be considered. The targeted construction of a blended care intervention could increase the existing potential of blended care intervention to promote physical activity. In the future, further high-quality studies should investigate which types of blended care intervention have the most beneficial effects in which target group and which intervention characteristics are most effective, by taking into account new evidence on behavior change.

## Supplementary Information

**Additional file 1:** PRISMA Checklist.

**Additional file 2:** Search terms.

**Additional file 3:** Risk of bias.

## Data Availability

Not applicable.
