# Supplementary material for: Blended Care Interventions to Promote Physical Activity: A Systematic Review of Randomized Controlled Trials
Source: Sports Med Open. 2022 Jul 30;8:100. doi: 10.1186/s40798-022-00489-w (PMC9339043; doi:10.1186/s40798-022-00489-w)
Supplement: Supplementary file 2 — Additional file 2: Search terms. [file 40798_2022_489_MOESM2_ESM.pdf]

Blended care interventions to promote physical activity - A systematic review of randomized controlled trials

Sports Medicine - Open

Vivien Hohberg [1] (vivien.hohberg@unibas.ch)

Reinhard Fuchs [2]

Markus Gerber [1]

David Künzler [2]

Sarah Paganini [2]

Oliver Faude [1]

1) Department of Sports, Exercise and Health, University of Basel, Switzerland

2) Department of Sport Psychology, Institute of Sports and Sport Science, University of Freiburg, Germany

Search component	Search string:<br>MEDLINE via Pubmed	Search string:<br>SportDiscus via EBSCO	Search string:<br>PsycInfo via EBSCO	Search string:<br>CINAHL via EBSCO	Search string:<br>Web of Science	Search string:<br>Cochrane CENTRAL
**Search component 1: Physical activity**	“PHYSICAL ACTIVITY” OR<br>“PHYSICAL EXERCISE” OR<br>SPORT OR<br>“PHYSICAL TRAINING” OR<br>“SPORT EXERCISE” OR<br>“SPORT ACTIVITY” OR<br>“EXERCISE THERAPY” OR<br>“FITNESS” OR<br>“PHYSICAL FITNESS” OR<br>“SPORT BEHAVIOR” OR<br>“PHYSICAL ACTIVITY PROMOTION” OR<br>“HEALTH BEHAVIOR”	“PHYSICAL ACTIVITY” OR<br>“PHYSICAL EXERCISE” OR<br>SPORT OR<br>“PHYSICAL TRAINING” OR<br>“SPORT EXERCISE” OR<br>“SPORT ACTIVITY” OR<br>“EXERCISE THERAPY” OR<br>“FITNESS” OR<br>“PHYSICAL FITNESS” OR<br>“SPORT BEHAVIOR” OR<br>“PHYSICAL ACTIVITY PROMOTION” OR<br>“HEALTH BEHAVIOR”	“PHYSICAL ACTIVITY” OR<br>“PHYSICAL EXERCISE” OR<br>SPORT OR<br>“PHYSICAL TRAINING” OR<br>“SPORT EXERCISE” OR<br>“SPORT ACTIVITY” OR<br>“EXERCISE THERAPY” OR<br>“FITNESS” OR<br>“PHYSICAL FITNESS” OR<br>“SPORT BEHAVIOR” OR<br>“PHYSICAL ACTIVITY PROMOTION” OR<br>“HEALTH BEHAVIOR”	“PHYSICAL ACTIVITY” OR<br>“PHYSICAL EXERCISE” OR<br>SPORT OR<br>“PHYSICAL TRAINING” OR<br>“SPORT EXERCISE” OR<br>“SPORT ACTIVITY” OR<br>“EXERCISE THERAPY” OR<br>“FITNESS” OR<br>“PHYSICAL FITNESS” OR<br>“SPORT BEHAVIOR” OR<br>“PHYSICAL ACTIVITY PROMOTION” OR<br>“HEALTH BEHAVIOR”	“PHYSICAL ACTIVITY” OR<br>“PHYSICAL EXERCISE” OR<br>SPORT OR<br>“PHYSICAL TRAINING” OR<br>“SPORT EXERCISE” OR<br>“SPORT ACTIVITY” OR<br>“EXERCISE THERAPY” OR<br>“FITNESS” OR<br>“PHYSICAL FITNESS” OR<br>“SPORT BEHAVIOR” OR<br>“PHYSICAL ACTIVITY PROMOTION” OR<br>“HEALTH BEHAVIOR”	“PHYSICAL ACTIVITY” OR<br>“PHYSICAL EXERCISE” OR<br>SPORT OR<br>“PHYSICAL TRAINING” OR<br>“SPORT EXERCISE” OR<br>“SPORT ACTIVITY” OR<br>“EXERCISE THERAPY” OR<br>“FITNESS” OR<br>“PHYSICAL FITNESS” OR<br>“SPORT BEHAVIOR” OR<br>“PHYSICAL ACTIVITY PROMOTION” OR<br>“HEALTH BEHAVIOR”
	AND	AND	AND	AND	AND	AND
**Search component 2ai: digital intervention**	INTERNET OR<br>“INTERNET INTERVENTION” OR<br>WEBSITE OR<br>“WEBSITE DELIVERED” OR<br>“WORLD WIDE WEB” OR<br>WEB-BASED OR<br>WEBBASED OR<br>INTERNETBASED OR<br>INTERNET-BASED OR<br>ONLINE OR<br>“ONLINE INTERVENTION” OR<br>COMPUTER OR<br>“COMPUTER-ASSISTED” OR<br>E-HEALTH OR<br>EHEALTH OR<br>SMARTPHONE OR<br>SMART-PHONE OR<br>CELLPHONE OR<br>CELL-PHONE OR<br>“MOBILE HEALTH” OR<br>MHEALTH OR<br>M-HEALTH OR<br>APP OR<br>APPLICATION OR<br>“MOBILE APPLICATION” OR<br>MOBILE-BASED OR<br>“MOBILE PHONE APPLICATION” OR<br>SMARTPHONE-BASED OR	INTERNET OR<br>“INTERNET INTERVENTION” OR<br>WEBSITE OR<br>“WEBSITE DELIVERED” OR<br>“WORLD WIDE WEB” OR<br>WEB-BASED OR<br>WEBBASED OR<br>INTERNETBASED OR<br>INTERNET-BASED OR<br>ONLINE OR<br>“ONLINE INTERVENTION” OR<br>COMPUTER OR<br>“COMPUTER-ASSISTED” OR<br>E-HEALTH OR<br>EHEALTH OR<br>SMARTPHONE OR<br>SMART-PHONE OR<br>CELLPHONE OR<br>CELL-PHONE OR<br>“MOBILE HEALTH” OR<br>MHEALTH OR<br>M-HEALTH OR<br>APP OR<br>APPLICATION OR<br>“MOBILE APPLICATION” OR<br>MOBILE-BASED OR<br>MOBILEBASED OR<br>“MOBILE PHONE APPLICATION” OR	INTERNET OR<br>“INTERNET INTERVENTION” OR<br>WEBSITE OR<br>“WEBSITE DELIVERED” OR<br>“WORLD WIDE WEB” OR<br>WEB-BASED OR<br>WEBBASED OR<br>INTERNETBASED OR<br>INTERNET-BASED OR<br>ONLINE OR<br>“ONLINE INTERVENTION” OR<br>COMPUTER OR<br>“COMPUTER-ASSISTED” OR<br>E-HEALTH OR<br>EHEALTH OR<br>SMARTPHONE OR<br>SMART-PHONE OR<br>CELLPHONE OR<br>CELL-PHONE OR<br>“MOBILE HEALTH” OR<br>MHEALTH OR<br>M-HEALTH OR<br>APP OR<br>APPLICATION OR<br>“MOBILE APPLICATION” OR<br>MOBILE-BASED OR<br>MOBILEBASED OR<br>“MOBILE PHONE APPLICATION” OR	INTERNET OR<br>“INTERNET INTERVENTION” OR<br>WEBSITE OR<br>“WEBSITE DELIVERED” OR<br>“WORLD WIDE WEB” OR<br>WEB-BASED OR<br>WEBBASED OR<br>INTERNETBASED OR<br>INTERNET-BASED OR<br>ONLINE OR<br>“ONLINE INTERVENTION” OR<br>COMPUTER OR<br>“COMPUTER-ASSISTED” OR<br>E-HEALTH OR<br>EHEALTH OR<br>SMARTPHONE OR<br>SMART-PHONE OR<br>CELLPHONE OR<br>CELL-PHONE OR<br>“MOBILE HEALTH” OR<br>MHEALTH OR<br>M-HEALTH OR<br>APP OR<br>APPLICATION OR<br>“MOBILE APPLICATION” OR<br>MOBILE-BASED OR<br>MOBILEBASED OR<br>“MOBILE PHONE APPLICATION” OR	INTERNET OR<br>“INTERNET INTERVENTION” OR<br>WEBSITE OR<br>“WEBSITE DELIVERED” OR<br>“WORLD WIDE WEB” OR<br>WEB-BASED OR<br>WEBBASED OR<br>INTERNETBASED OR<br>INTERNET-BASED OR<br>ONLINE OR<br>“ONLINE INTERVENTION” OR<br>COMPUTER OR<br>“COMPUTER-ASSISTED” OR<br>E-HEALTH OR<br>EHEALTH OR<br>SMARTPHONE OR<br>SMART-PHONE OR<br>CELLPHONE OR<br>CELL-PHONE OR<br>“MOBILE HEALTH” OR<br>MHEALTH OR<br>M-HEALTH OR<br>APP OR<br>APPLICATION OR<br>“MOBILE APPLICATION” OR<br>MOBILE-BASED OR<br>MOBILEBASED OR<br>“MOBILE PHONE APPLICATION” OR	INTERNET OR<br>“INTERNET INTERVENTION” OR<br>WEBSITE OR<br>“WEBSITE DELIVERED” OR<br>“WORLD WIDE WEB” OR<br>WEB-BASED OR<br>WEBBASED OR<br>INTERNETBASED OR<br>INTERNET-BASED OR<br>ONLINE OR<br>“ONLINE INTERVENTION” OR<br>COMPUTER OR<br>“COMPUTER-ASSISTED” OR<br>E-HEALTH OR<br>EHEALTH OR<br>SMARTPHONE OR<br>SMART-PHONE OR<br>CELLPHONE OR<br>CELL-PHONE OR<br>“MOBILE HEALTH” OR<br>MHEALTH OR<br>M-HEALTH OR<br>APP OR<br>APPLICATION OR<br>“MOBILE APPLICATION” OR<br>MOBILE-BASED OR<br>MOBILEBASED OR<br>“MOBILE PHONE APPLICATION” OR

Search component	Search string: MEDLINE via Pubmed	Search string: SportDiscus via EBSCO	Search string: PsycInfo via EBSCO	Search string: CINAHL via EBSCO	Search string: Web of Science	Search string: Cochrane CENTRAL
	“MOBILE DEVICE” OR “DIGITAL HEALTHCARE” OR INFORMATION COMMUNICATION TECHNOLOG* OR “TELEMEDICINE” OR IMI	SMARTPHONE-BASED OR “MOBILE DEVICE” OR “DIGITAL HEALTHCARE” OR INFORMATION COMMUNICATION TECHNOLOG* OR “TELEMEDICINE” OR IMI	SMARTPHONE-BASED OR “MOBILE DEVICE” OR “DIGITAL HEALTHCARE” OR INFORMATION COMMUNICATION TECHNOLOG* OR “TELEMEDICINE” OR IMI	SMARTPHONE-BASED OR “MOBILE DEVICE” OR “DIGITAL HEALTHCARE” OR INFORMATION COMMUNICATION TECHNOLOG* OR “TELEMEDICINE” OR IMI	SMARTPHONE-BASED OR “MOBILE DEVICE” OR “DIGITAL HEALTHCARE” OR INFORMATION COMMUNICATION TECHNOLOG* OR “TELEMEDICINE” OR IMI	SMARTPHONE-BASED OR “MOBILE DEVICE” OR “DIGITAL HEALTHCARE” OR INFORMATION COMMUNICATION TECHNOLOG* OR “TELEMEDICINE” OR IMI
	AND	AND	AND	AND	AND	AND
**Search component 2aii: therapist-guided intervention**	FACE-TO-FACE OR “FACE TO FACE” OR F2F OR VIDEOCONFERENCING OR VIDEO-CONFERENCING OR TELEPHONE OR VIDEO OR “PERSONAL EMAIL” OR “PERSONAL E-MAIL” OR “TAILORED EMAIL” OR “TAILORED E-MAIL” OR CENTER-BASED OR INPATIENT OR OUTPATIENT OR COUNSELING OR “RESIDENTIAL TREATMENT”	FACE-TO-FACE OR “FACE TO FACE” OR F2F OR VIDEOCONFERENCING OR VIDEO-CONFERENCING OR TELEPHONE OR VIDEO OR “PERSONAL EMAIL” OR “PERSONAL E-MAIL” OR “TAILORED EMAIL” OR “TAILORED E-MAIL” OR CENTERBASED OR CENTER-BASED OR INPATIENT OR OUTPATIENT OR COUNSELING OR “RESIDENTIAL TREATMENT”	FACE-TO-FACE OR “FACE TO FACE” OR F2F OR VIDEOCONFERENCING OR VIDEO-CONFERENCING OR TELEPHONE OR VIDEO OR “PERSONAL EMAIL” OR “PERSONAL E-MAIL” OR “TAILORED EMAIL” OR “TAILORED E-MAIL” OR CENTERBASED OR CENTER-BASED OR INPATIENT OR OUTPATIENT OR COUNSELING OR “RESIDENTIAL TREATMENT”	FACE-TO-FACE OR “FACE TO FACE” OR F2F OR VIDEOCONFERENCING OR VIDEO-CONFERENCING OR TELEPHONE OR VIDEO OR “PERSONAL EMAIL” OR “PERSONAL E-MAIL” OR “TAILORED EMAIL” OR “TAILORED E-MAIL” OR CENTERBASED OR CENTER-BASED OR INPATIENT OR OUTPATIENT OR COUNSELING OR “RESIDENTIAL TREATMENT”	FACE-TO-FACE OR “FACE TO FACE” OR F2F OR VIDEOCONFERENCING OR VIDEO-CONFERENCING OR TELEPHONE OR VIDEO OR “PERSONAL EMAIL” OR “PERSONAL E-MAIL” OR “TAILORED EMAIL” OR “TAILORED E-MAIL” OR CENTERBASED OR CENTER-BASED OR INPATIENT OR OUTPATIENT OR COUNSELING OR “RESIDENTIAL TREATMENT”	FACE-TO-FACE OR “FACE TO FACE” OR F2F OR VIDEOCONFERENCING OR VIDEO-CONFERENCING OR TELEPHONE OR VIDEO OR “PERSONAL EMAIL” OR “PERSONAL E-MAIL” OR “TAILORED EMAIL” OR “TAILORED E-MAIL” OR CENTERBASED OR CENTER-BASED OR INPATIENT OR OUTPATIENT OR COUNSELING OR “RESIDENTIAL TREATMENT”
	OR	OR	OR	OR	OR	OR
**Search component 2b: Blended care intervention**	BLENDED OR “BLENDED INTERVENTION” OR “BLENDED CARE” OR “BLENDED HEALTH” OR “BLENDED HEALTH CARE” OR “BLENDED PROGRAM” OR “BLENDED TREATMENT” OR “BLENDED CARE TREATMENT” OR “COMBINED INTERVENTION” OR “COMBINED TREATMENT” OR	BLENDED OR “BLENDED INTERVENTION” OR “BLENDED CARE” OR “BLENDED HEALTH” OR “BLENDED HEALTH CARE” OR “BLENDED PROGRAM” OR “BLENDED TREATMENT” OR “BLENDED CARE TREATMENT” OR “COMBINED INTERVENTION” OR “COMBINED TREATMENT” OR	BLENDED OR “BLENDED INTERVENTION” OR “BLENDED CARE” OR “BLENDED HEALTH” OR “BLENDED HEALTH CARE” OR “BLENDED PROGRAM” OR “BLENDED TREATMENT” OR “BLENDED CARE TREATMENT” OR “COMBINED INTERVENTION” OR “COMBINED TREATMENT” OR	BLENDED OR “BLENDED INTERVENTION” OR “BLENDED CARE” OR “BLENDED HEALTH” OR “BLENDED HEALTH CARE” OR “BLENDED PROGRAM” OR “BLENDED TREATMENT” OR “BLENDED CARE TREATMENT” OR “COMBINED INTERVENTION” OR “COMBINED TREATMENT” OR	BLENDED OR “BLENDED INTERVENTION” OR “BLENDED CARE” OR “BLENDED HEALTH” OR “BLENDED HEALTH CARE” OR “BLENDED PROGRAM” OR “BLENDED TREATMENT” OR “BLENDED CARE TREATMENT” OR “COMBINED INTERVENTION” OR “COMBINED TREATMENT” OR	BLENDED OR “BLENDED INTERVENTION” OR “BLENDED CARE” OR “BLENDED HEALTH” OR “BLENDED HEALTH CARE” OR “BLENDED PROGRAM” OR “BLENDED TREATMENT” OR “BLENDED CARE TREATMENT” OR “COMBINED INTERVENTION” OR “COMBINED TREATMENT” OR

**Search component**	**Search string: MEDLINE via Pubmed**	**Search string: SportDiscus via EBSCO**	**Search string: PsycInfo via EBSCO**	**Search string: CINAHL via EBSCO**	**Search string: Web of Science**	**Search string: Cochrane CENTRAL**
	"COMBINED CARE" OR "COMBINED PROGRAM" OR "MIXED HEALTH" OR "MIXED HEALTH CARE" OR "MIXED CARE" OR "MIXED TREATMENT" OR "MIXED INTERVENTION"	"COMBINED CARE" OR "COMBINED PROGRAM" OR "MIXED HEALTH" OR "MIXED HEALTH CARE" OR "MIXED CARE" OR "MIXED TREATMENT" OR "MIXED INTERVENTION"	"COMBINED CARE" OR "COMBINED PROGRAM" OR "MIXED HEALTH" OR "MIXED HEALTH CARE" OR "MIXED CARE" OR "MIXED TREATMENT" OR "MIXED INTERVENTION"	"COMBINED CARE" OR "COMBINED PROGRAM" OR "MIXED HEALTH" OR "MIXED HEALTH CARE" OR "MIXED CARE" OR "MIXED TREATMENT" OR "MIXED INTERVENTION"	"COMBINED CARE" OR "COMBINED PROGRAM" OR "MIXED HEALTH" OR "MIXED HEALTH CARE" OR "MIXED CARE" OR "MIXED TREATMENT" OR "MIXED INTERVENTION"	"COMBINED CARE" OR "COMBINED PROGRAM" OR "MIXED HEALTH" OR "MIXED HEALTH CARE" OR "MIXED CARE" OR "MIXED TREATMENT" OR "MIXED INTERVENTION"
	AND	AND	AND	AND	AND	AND
**Search component 3: Randomized controlled trial**	RANDOMI* CONTROLLED TRIAL OR RANDOMI* CONTROLLED OR RCT OR RANDOMI* OR "INTERVENTION STUDY" OR RANDOM* CLINICAL TRIAL OR CONTROL* CLINICAL TRIAL	RANDOMI* CONTROLLED TRIAL OR RANDOMI* CONTROLLED OR RCT OR RANDOMI* OR "INTERVENTION STUDY" OR RANDOM* CLINICAL TRIAL OR CONTROL* CLINICAL TRIAL	RANDOMI* CONTROLLED TRIAL OR RANDOMI* CONTROLLED OR RCT OR RANDOMI* OR "INTERVENTION STUDY" OR RANDOM* CLINICAL TRIAL OR CONTROL* CLINICAL TRIAL	RANDOMI* CONTROLLED TRIAL OR RANDOMI* CONTROLLED OR RCT OR RANDOMI* OR "INTERVENTION STUDY" OR RANDOM* CLINICAL TRIAL OR CONTROL* CLINICAL TRIAL	RANDOMI* CONTROLLED TRIAL OR RANDOMI* CONTROLLED OR RCT OR RANDOMI* OR "INTERVENTION STUDY" OR RANDOM* CLINICAL TRIAL OR CONTROL* CLINICAL TRIAL	RANDOMI* CONTROLLED TRIAL OR RANDOMI* CONTROLLED OR RCT OR RANDOMI* OR "INTERVENTION STUDY" OR RANDOM* CLINICAL TRIAL OR CONTROL* CLINICAL TRIAL
