# Supplementary material for: Blended Care Interventions to Promote Physical Activity: A Systematic Review of Randomized Controlled Trials
Source: Sports Med Open. 2022 Jul 30;8:100. doi: 10.1186/s40798-022-00489-w (PMC9339043; doi:10.1186/s40798-022-00489-w)
Supplement: Supplementary file 3 — Additional file 3: Risk of bias. [file 40798_2022_489_MOESM3_ESM.pdf]

Blended care interventions to promote physical activity - A systematic review of randomized controlled trials

Sports Medicine - Open

Vivien Hohberg [1] (vivien.hohberg@unibas.ch)

Reinhard Fuchs [2]

Markus Gerber [1]

David Künzler [2]

Sarah Paganini [2]

Oliver Faude [1]

1) Department of Sports, Exercise and Health, University of Basel, Switzerland

2) Department of Sport Psychology, Institute of Sports and Sport Science, University of Freiburg, Germany

Table S2: Risk of bias of blended care intervention to promote physical activity

Study ID	Experimental	Comparator	Outcome	Weight	Randomization process	Deviations from intended interve	Missing outcome data	Measurement of the outcome	Selection of the reported result	Overall
Albright	Blended Care	DI	Physical Activity	1	+	+	+	+	+	+
Alley	Blended Care	WL, DI	Physical Activity	1	?	?	+	?	+	!
Anderson	Blended Care	TAU	Physical Activity	1	+	+	?	+	?	!
Broekhuizen	Blended Care	TAU	Physical Activity	1	+	+	+	?	+	+
Christian	Blended Care	TAU	Physical Activity	1	+	+	+	?	?	!
Collins	Blended Care	TAU	Physical Activity	1	+	+	+	+	+	+
Crane	Blended Care	WL	Physical Activity	1	+	+	+	?	+	+
Duncan	Blended Care	WL	Physical Activity	1	+	+	+	+	+	+
Fischer	Blended Care	DI	Physical Activity	1	+	+	–	+	+	!
Glasgow	Blended Care	TAU, DI	Physical Activity	1	?	+	+	?	+	!
McDermott	Blended Care	TAU	Physical Activity	1	+	+	?	+	+	!
Morgan	Blended Care	TAU	Physical Activity	1	+	?	+	+	+	+
Mouton	Blended Care	WL, DI, TGI	Physical Activita	1	+	+	?	?	+	!
Partridge	Blended Care	DI	Physical Activity	1	+	+	+	?	+	+
Plotnikoff	Blended Care	WL	physical activity	1	+	+	+	+	+	+
Richardson	Blended Care	DI	physical activity	1	?	+	?	+	+	!
Rubinstein	Blended Care	TAU	physical activity	1	+	+	+	?	+	+
Schaller	Blended Care	TAU	physical activity	1	+	+	+	?	+	+
Sniehotta	Blended Care	DI	physical activity	1	+	+	+	+	+	+
Steele	Blended Care	DI, TGI	physical activity	1	+	+	+	?	+	+
Torbjørnsen	Blended Care	TAU, DI	physical activity	1	+	+	+	?	+	!
Turner	Blended Care	TAU	physical activity	1	+	+	+	?	+	!
van der Weegen	Blended Care	TAU, TGI	physical activity	1	+	+	+	+	+	+
Wilbur	Blended Care	TGI	physical activity	1	+	?	+	+	+	+
Wylie-Rosett	Blended Care	TAU	physical activity	1	?	?	?	?	+	!

+	Low risk
?	Some concerns
–	High risk

DI: digital intervention, TAU: treatment as usual, TGI: therapist-guided intervention, WL: Waiting list

Sterne JA, Savović J, Page MJ, Elbers RG, Blencowe NS, Boutron I, et al. RoB 2: a revised tool for assessing risk of bias in randomised trials. BMJ. 2019;366.
